# Impact of Shift Work and Job Category on Lifestyle Factors and Readiness to Change Among Hospital Workers: A Case-Control Study

**DOI:** 10.7759/cureus.72516

**Published:** 2024-10-28

**Authors:** Prathamesh H Kamble, Akshat Agarwal, Ashwini Hedaoo, Mrunal Phatak

**Affiliations:** 1 Department of Physiology, All India Institute of Medical Sciences Nagpur, Nagpur, IND

**Keywords:** hospital workers, lifestyle factors, nurses, shift work, support staff

## Abstract

Background: Shift work is essential in health care because of the need for 24-hour services but it is associated with adverse health outcomes, including disrupted circadian rhythms, poor sleep, unhealthy dietary habits, and increased stress. These effects may differ across job categories, such as nursing officers and hospital support staff, owing to varying physical and psychological demands. Limited research exists on how shift work impacts these groups differently, particularly regarding readiness to change unhealthy lifestyle behaviors.

Objectives: This study aims to assess and compare lifestyle factors across six domains - nutrition, physical activity, sleep, stress, social relationships, and addictions - between hospital support staff and nursing officers with rotating shifts versus fixed daytime duties. It also aims to evaluate the association between readiness to change lifestyle patterns and work type and determine the influence of job category and shift type on lifestyle parameters after adjusting for confounders such as demographics and body composition.

Methodology: A case-control study was conducted at All India Institute of Medical Sciences (AIIMS) Nagpur from December 2023 to June 2024. The study involved 327 participants (165 cases and 162 controls) comprising nurses and hospital support staff, aged 21-45 years. The case group included 83 nurses and 84 hospital support staff working rotating shifts for at least three years, while the control group consisted of 81 nurses and 81 staff members with fixed daytime schedules. General assessments, including demographics, body composition (InBody 770), and lifestyle assessments across nutrition, physical activity, sleep (Pittsburgh Sleep Quality Index; PSQI), stress (Perceived Stress Scale; PSS-10), social connectivity (Social Support Questionnaire), and alcohol use (a modified version of the 10-item Alcohol Use Disorders Identification Test (AUDIT-C)) were performed. Readiness to change lifestyle behaviors was assessed using the stages of the change model.

Results: Shift workers had a significantly higher body weight (p = 0.030), larger waist circumference (p = 0.029), and higher calorie intake (p = 0.043) than non-shift workers. They also exhibited lower cardiovascular fitness (p = 0.021) and reduced water intake (p = 0.043). Among the nursing officers, shift workers had significantly poorer sleep quality (p = 0.003) and higher calorie intake (p = 0.046). Stress levels were paradoxically lower among shift nurses (p = 0.025) but not among support staff. Readiness to change lifestyle behaviors did not differ significantly between shift and non-shift workers across all domains. Logistic regression showed that sleep quality was significantly associated with shift work among nursing officers (odds ratio (OR): 6.503, p = 0.038), while no significant associations were found for other lifestyle parameters.

Conclusion: This study highlights the adverse effects of shift work on body composition, calorie intake, cardiovascular fitness, and sleep quality among hospital workers, particularly nursing officers. Despite these health risks, readiness to change lifestyle behaviors was similar between shift and non-shift workers, suggesting that shift work may not directly influence the motivation for lifestyle changes. These findings underscore the need for tailored interventions targeting specific health challenges of shift workers to improve their overall well-being and productivity.

## Introduction

Shift work, especially in health care, is becoming increasingly common because of the need for 24-hour services. Rotating shift work is associated with several adverse health outcomes, including disrupted circadian rhythms, altered sleep patterns, and unhealthy lifestyle behaviors such as poor nutrition, inadequate physical activity, and increased stress [1]. These effects may vary depending on job categories and roles, such as hospital support staff (housekeeping, porters, security guards, technicians, and maintenance staff) and nursing officers, who experience different levels of physical and psychological demands during their shifts. Understanding how shift work and job categories influence lifestyle factors and readiness to change behaviors is crucial for developing interventions to improve the well-being of hospital workers.

Previous research has established that shift work can negatively affect health and lifestyle parameters, particularly among healthcare professionals [1-3]. However, there is limited evidence comparing the impact of shift work on different categories of hospital staff, such as nursing officers versus support staff, and how these factors may influence readiness to change unhealthy lifestyle habits. Given the growing concerns regarding occupational health in hospital settings, it is essential to assess the relationship between job type (shift vs. non-shift) and key lifestyle domains, including nutrition, physical exercise, sleep, stress, social relationships, and addiction.

Hospital workers often face the dual burden of maintaining their own health while attending to the health of others. Both nursing officers and support staff are exposed to the demands of shift work, but the physical and emotional strains may vary between these job roles. It is important to understand how these differences affect lifestyle factors and the readiness to adopt healthier habits. Additionally, by adjusting for potential confounders such as age, sex, and body composition, this study can provide a more nuanced understanding of the specific factors that contribute to lifestyle changes in these populations. This will guide the development of more effective and tailored interventions to mitigate the negative effects of shift work.

This study aims to fill a critical gap in the literature by simultaneously comparing lifestyle factors across multiple domains between hospital support staff and nursing officers with rotating shifts versus fixed daytime duties. Unlike previous studies which often focus on a single job category or specific health outcomes, this study explores a broader range of lifestyle parameters and examines the readiness to change across both groups. Furthermore, the study incorporates the analysis of multiple confounding factors, providing a comprehensive perspective on the influence of job type and shift work on lifestyle behaviors. This could lead to more specific and actionable recommendations for improving the health and well-being of hospital workers across different roles.

By addressing both job categories and adjusting for demographic and body composition factors, this study offers a novel approach to understanding the complex relationship between shift work and lifestyle health. These findings will be instrumental in designing workplace health interventions tailored to different job roles, ultimately enhancing the health and productivity of hospital staff.

## Materials and methods

This case-control study was conducted from December 2023 to June 2024 at All India Institute of Medical Sciences (AIIMS) Nagpur, involving nurses and hospital support staff, including housekeeping, porters, security guards, technicians, and maintenance staff from the hospital.

Nurses and hospital support staff, at AIIMS Nagpur, work under two types of schedules: rotating shift duty and fixed daytime duty. The fixed daytime schedule runs from 9 AM to 5 PM, with Sundays off. The rotating shift schedule consists of two days of morning duty, two days of afternoon duty, and two days of night duty, followed by two days off. In this study, we included workers following the rotating shift pattern as the case group and those on the fixed daytime schedule as the control group.

Institutional ethics committee approval was obtained from AIIMS Nagpur (IEC No. : IEC/PHARMAC/2022/432) before the start of the study. Nurses and hospital support staff were informed about the study and invited to participate via text messages and WhatsApp announcements. A total of 165 cases and 162 controls met the inclusion and exclusion criteria and agreed to participate. The case group consisted of 83 nurses and 84 hospital support staff who had been working rotating shifts for at least three years, while the control group comprised 81 nurses and 81 hospital support staff who had been on a fixed daytime duty schedule for the same period. The literature suggests that short-term shift work, less than six months, can impact lifestyle factors such as sleep, diet, and physical activity [4]. However, long-term shift work (beyond six months) shows stronger associations with significant lifestyle changes and adverse health outcomes [1-3]. In the present study, a three-year duration was chosen, as all nurses and hospital support staff were recruited four years ago. Considering their initial departmental rotations and the time needed for orientation, three years was set as the minimum inclusion criterion for both shift and non-shift workers.

Eligible participants were aged between 21 and 45 years, of either sex and provided written consent. Individuals who were physically handicapped or unable to perform physical activity tests were excluded from the study.

Written informed consent was obtained from all participants upon enrollment in the study. General and lifestyle assessments were also conducted. For the general assessment, demographic data were collected, including details about shift work (shift type, total working hours, weekly offs), as well as medical history, such as current illnesses, past medical conditions requiring hospitalization, personal history, family history, and menstrual and obstetric history for female participants. During the physical examination, pulse and blood pressure were recorded, and measurements of height, waist circumference, and waist-to-height ratio were also recorded according to the World Health Organization (WHO) standards. Body composition was assessed using the InBody 770 multi-frequency bioelectrical impedance analysis (BIA) device (InBody Co., Ltd., Seoul, Korea). It provided data on body weight, body fat percentage, visceral fat percentage, subcutaneous fat levels (whole body, trunk, arms, and legs), skeletal muscle percentage, and body mass index (BMI).

Among the lifestyle assessments, nutrition was evaluated through a 24-hour dietary recall conducted over the past 15 days, using an online food diary to record each food item consumed along with its approximate quantity. Participants were shown standard spoons and bowls to estimate intake, and the total energy expenditure and water intake were calculated using the Nutrinix software. The total calorie intake (in kilocalories) was recorded.

Physical activity was assessed following the Fit India Guidelines, using a scoring system (Fit India reference), which included components such as flexibility (V Sit Reach Test), muscular strength (Partial Curl Up - 30 seconds), muscular endurance (push-ups for males, modified push-ups for females), body composition (BMI), cardiovascular fitness (2 km run/walk), and static balance (Flamingo balance test). An age- and sex-standardized scoring system for the Indian population was applied, as per the Fit India guidelines [5].

Sleep quality was measured using the Pittsburgh Sleep Quality Index (PSQI), a self-rated questionnaire that assesses sleep quality and disturbances over a one-month period. The PSQI comprises 19 individual items that generate seven component scores: subjective sleep quality, sleep latency, sleep duration, habitual sleep efficiency, sleep disturbances, use of sleep medication, and daytime dysfunction, which are summed to yield a global score [6]. It is freely available for use in academic and clinical research without the need to contact the Office of Technology Management at the University of Pittsburgh.

Stress levels were evaluated using the Perceived Stress Scale (PSS-10), which is a 10-item questionnaire that measures how individuals perceive stress in their lives. Each item is rated on a 5-point scale from “never” (0) to “almost always” (4), with positively worded items reverse-scored. Higher scores indicate higher levels of perceived stress, with scores of approximately 13 being considered average [7]. The tool is available for non-commercial academic and clinical research without the need for explicit permission from the authors.

Social connectivity was assessed using the 27-item Social Support Questionnaire, which measures perceptions of social support and satisfaction, asking participants to list individuals who provide support and rate their satisfaction with them. This questionnaire tool is available for non-commercial academic and clinical research without requiring formal permission from the authors [8].

Alcohol consumption was evaluated using the AUDIT-C for alcohol use, a modified version of the 10-item Alcohol Use Disorders Identification Test developed by the WHO [9].

The stage of readiness to change for each lifestyle domain was assessed based on the five stages of readiness to change (pre-contemplation, contemplation, preparation, action, and maintenance) as outlined by Weiner et al., 2009 [10].

Statistical analysis

Descriptive statistics were calculated for continuous variables (e.g., body weight and calorie intake) and categorical variables (e.g., sex and work type) to summarize the data. Independent t-tests were used to compare continuous variables between shift and non-shift workers. Chi-square tests with Yates correction were used to assess the association between readiness to change lifestyle patterns and work type. Multiple linear regression was performed to examine the relationship between total calorie intake and factors such as work type, job category, age, and sex. Adjusted odds ratios (ORs) with 95% confidence intervals (CIs) were calculated using logistic regression to evaluate the associations between shift work and various lifestyle parameters. Statistical significance was set at p <0.05, and all analyses were conducted using SPSS version 25.0 (IBM Corp., Armonk, NY).

## Results

Table 1 presents the descriptive statistics for the various characteristics of workers with non-shift and shift job profiles. The differences in age, sex, and blood pressure between the two groups were not statistically significant, indicating that the baseline characteristics were comparable. Shift workers tend to have higher body weight (64.79 ± 8.52 kg vs. 57.14 ± 9.74 kg, p = 0.030) and larger waist circumference (34.40 ± 1.72 cm vs. 32.63 ± 2.42 cm, p = 0.029) compared to non-shift workers. The shift workers also consumed more calories (p = 0.043) and drank less water (p = 0.043). In addition, the cardiovascular fitness was significantly lower (p = 0.021). No significant differences were found in sleep duration, stress levels, or social connectivity between the groups. These results indicate that shift workers may be at greater risk of weight gain and reduced cardiovascular health (Table 1).

**Table 1:** Descriptive statistics for various characteristics of hospital support staff with non-shift and shift job profiles Data represent mean and standard deviation. SBP, systolic blood pressure; DBP, diastolic blood pressure; BMI, body mass index; PSQI, Pittsburgh Sleep Quality Index; PSS, Perceived Stress Scale. *p-Value obtained using chi-square test; other p-values obtained using t-test for independent samples.

Category	Parameter		Non-shift	Shift	p-Value
Demographics	Age (years)		27.53 ± 4.49	31.07 ± 5.11	0.054
	Sex*	Male	(73.3%)	(53.3%)	0.256
		Female	(26.7%)	(46.7%)	
	SBP (mmHg)		113.2 ± 10.8	118.13 ± 13.42	0.277
	DBP (mmHg)		74.87 ± 6.31	76.47 ± 8.66	0.568
	Waist circumference (cm)		32.63 ± 2.42	34.40 ± 1.72	0.029
	Waist-to-height ratio		0.50 ± 0.05	0.52 ± 0.04	0.356
Body composition analysis	Body weight (kg)		57.14 ± 9.74	64.79 ± 8.52	0.030
	Body fat		26.92 ± 6.66	27.64 ± 5.37	0.747
	Visceral fat percentage		5.83 ± 3.74	6.33 ± 3.11	0.694
	Whole body fat level		19.79 ± 6.04	21.37 ± 5.97	0.477
	Trunk		17.25 ± 5.98	18.69 ± 5.11	0.484
	Arms		31.74 ± 11.74	33.48 ± 10.59	0.673
	Legs		29.65 ± 8.82	31.03 ± 8.47	0.664
	Skeletal muscle ratio - whole body		29.50 ± 4.10	28.89 ± 3.93	0.682
	Skeletal muscle ratio - trunk		22.97 ± 3.69	22.56 ± 3.52	0.756
	Skeletal muscle ratio - arms		35.31 ± 5.95	32.93 ± 5.98	0.284
	Skeletal muscle ratio - legs		44.86 ± 6.37	43.86 ± 6.18	0.666
	BMI (kg/m^2^)		22.17 ± 3.67	23.35 ± 2.45	0.306
Lifestyle assessment					
Nutrition	Total calories intake		2238.55 ±559.87	2618.93 ± 411.37	0.043
	Water intake in liters		4.43 ± 2.43	2.82 ± 1.5	0.043
Physical fitness	Flexibility		4.67 ± 2.69	4.67 ± 2.64	1.000
	Muscle strength crunches		1.13 ± 0.35	1.07 ± 0.26	0.559
	Muscle endurance push		3.13 ± 1.30	3.27 ± 0.88	0.745
	Cardio fitness		4.13 ± 2.92	2.00 ± 1.69	0.021
	Flamingo balance test score		1.13 ± 1.36	2.20 ± 3.08	0.229
Sleep	Weekday hours of sleep		7.17 ± 1.26	6.57 ± 1.39	0.226
	Weekend hours of sleep		9.00 ± 1.74	8.07 ± 1.40	0.117
	PSQ - Sleep		7.47 ± 2.50	7.33 ± 2.23	0.879
	PSQI - Sleep		4.73 ± 1.94	5.40 ± 2.64	0.438
Stress	PSS - Stress		15.20 ± 7.49	17.33 ± 8.05	0.459
Social connectivity	Social connectivity score		4.20 ± 1.81	4.33 ± 1.85	0.843

The descriptive statistics in Table 2 compare the characteristics of nursing officers with the non-shift and shift job profiles. Shift nursing officers had a significantly higher calorie intake (p = 0.046), lower cardiovascular fitness (p = 0.042), and poorer sleep quality (p = 0.003) than non-shift workers. However, shift workers reported lower stress levels (p = 0.025). Other factors, such as body composition, muscle strength, and social connectivity, showed no significant differences between the two groups (Table 2).

**Table 2:** Descriptive statistics for various characteristics of nursing officers with non-shift and shift job profiles Data represent mean and standard deviation. SBP, systolic blood pressure; DBP, diastolic blood pressure; BMI, body mass index; PSQI, Pittsburgh Sleep Quality Index; PSS, Perceived Stress Scale. *p-Value obtained using chi-square test; other p-values were obtained using t-test for independent samples.

Category	Parameter	Non-shift	Shift	p-Value
Demographics	Age (years)	27.53 ± 1.81	26.33 ± 2.13	0.107
	Sex*			
	Male	6 (40%)	4 (26.7%)	0.439
	Female	9 (60%)	11 (73.3%)	
Physical examination	Pulse/min	84.07 ± 9.38	89.87 ± 13.84	0.190
	SBP (mmHg)	113.67 ± 17.81	115.6 ± 14.57	0.747
	DBP (mmHg)	76.8 ± 12.08	77.07 ± 7.97	0.944
	Height (cm)	161.53 ± 11.75	161.33 ± 8.62	0.958
	Waist circumference (cm)	33.10 ± 2.90	34.73 ± 3.77	0.194
	Waist-to-height ratio	0.51 ± 0.06	0.54 ± 0.05	0.221
Body composition analysis	Body weight (kg)	59.95 ± 17.39	62.83 ± 13.95	0.620
	Body fat	29.41 ± 6.13	30.45 ± 5.78	0.636
	Visceral fat percentage	4.90 ± 4.32	6.70 ± 3.61	0.226
	Whole body fat level	23.42 ± 5.9	23.98 ± 6.50	0.807
	Trunk	20.49 ± 5.55	21.51 ± 6.21	0.639
	Arms	38.98 ± 11.07	38.53 ± 11.51	0.915
	Legs	35.02 ± 9.13	35.59 ± 9.82	0.871
	Skeletal muscle ratio - whole body	27.12 ± 4.36	27.10 ± 3.800	0.989
	Skeletal muscle ratio - trunk	21.73 ± 3.6	21.09 ± 2.99	0.601
	Skeletal muscle ratio - arms	30.74 ± 5.53	30.86 ± 6.46	0.957
	Skeletal muscle ratio - legs	40.25 ± 6.77	71.21 ± 119.04	0.323
	Resting metabolism	1151.04 ± 352.55	1371.53 ± 265.8	0.063
	BMI (kg/m^2^)	22.44 ± 3.81	24.00 ± 3.75	0.268
	Body age (years)	32.80 ± 10.09	37.00 ± 9.93	0.260
Lifestyle assessment (nutrition)	Total calories intake	2004.27 ± 698.79	2494.57 ± 581.45	0.046
	Water intake in liters	2.50 ± 0.94	2.10 ± 0.69	0.195
Physical activity	Flexibility	6.13 ± 1.68	5.33 ± 2.38	0.297
	Muscle strength crunches	1.33 ± 0.82	1.27 ± 0.46	0.785
	Muscle endurance push	3.13 ± 1.41	3.00 ± 1.00	0.767
	Cardio fitness	2.93 ± 2.63	1.40 ± 0.91	0.042
	Flamingo balance test score	1.33 ± 1.54	1.20 ± 1.57	0.816
Sleep	Weekday hours of sleep	7.23 ± 0.96	6.83 ± 0.84	0.235
	Weekend hours of sleep	9.03 ± 1.56	8.37 ± 1.34	0.221
	PSQ - Sleep	7.40 ± 1.84	7.20 ± 1.42	0.742
	PSQI - Sleep	6.00 ± 2.24	3.20 ± 2.46	0.003
Stress	PSS - Stress	17.07 ± 7.51	11.27 ± 5.81	0.025
Social connectivity	Social connectivity score	5.00 ± 1.20	4.51 ± 1.93	0.407

Tables 3, 4 display the association between readiness to change various lifestyle patterns and the type of work among hospital support staff and nursing officers, respectively, using the chi-square test with Yates correction. In both groups, most workers were in the pre-contemplation stage for all domains of lifestyle, with some shift workers progressing to the action and maintenance stages. In both hospital support staff and nursing officers, there were no statistically significant differences in readiness to change various lifestyle patterns between shift and non-shift workers across all domains (nutrition, physical activity, sleep, stress, social connectivity, and addictions). While shift workers generally showed higher percentages in the pre-contemplation stage in some lifestyle areas, such as nutrition and sleep, these differences were not statistically significant, indicating that type of work (shift vs. non-shift) did not have a clear impact on readiness to change lifestyle behaviors (Tables 3, 4).

**Table 3:** Association of readiness to change in various lifestyle patterns with type of work among hospital support staff *Obtained using chi-square test with Yates correction.

Patterns of lifestyle change	Readiness to change	Type of work		p-Value*
		Non-shift (N = 81)	Shift (N = 84)	
Nutrition	Pre-contemplation	54 (67%)	62 (74%)	0.133
	Contemplation	22 (27%)	0	
	Preparation	5 (6%)	5 (6%)	
	Action	0	6 (7%)	
	Maintenance	0	11 (13%)	
Physical activity	Pre-contemplation	22 (27%)	17 (20%)	0.976
	Contemplation	22 (27%)	22 (27%)	
	Preparation	22 (27%)	22 (27%)	
	Action	5 (7%)	11 (13%)	
	Maintenance	11 (13%)	11 (13%)	
Sleep	Pre-contemplation	65 (80%)	39 (47%)	0.186
	Contemplation	16 (20%)	28 (33%)	
	Preparation	0	6 (7%)	
	Maintenance	0	11 (13%)	
Stress	Pre-contemplation	38 (47%)	39 (47%)	0.525
	Contemplation	22 (27%)	22 (27%)	
	Preparation	16 (20%)	11 (13%)	
	Action	0	11 (13%)	
	Maintenance	5 (17%)	0	
Social connectivity	Pre-contemplation	70 (87%)	56 (67%)	0.274
	Contemplation	11 (13%)	17 (20%)	
	Maintenance	0	11 (13%)	
Addictions	Pre-contemplation	4	3	0.558
	Contemplation	0	1	
	Action	1	1	
	Maintenance	1	0	

**Table 4:** Association of readiness to change in various lifestyle patterns with type of work among nursing officers *Obtained using chi-square test with Yates correction.

Patterns of lifestyle change	Readiness to change	Type of work		p-Value*
		Non-shift (N = 81)	Shift (N = 83)	
Nutrition	Pre-contemplation	11 (13%)	33 (40%)	0.298
	Contemplation	22 (27%)	11 (13%)	
	Preparation	38 (47%)	22 (27%)	
	Action	11 (13%)	17 (20%)	
Physical exercise	Pre-contemplation	5 (7%)	28 (33%)	0.119
	Contemplation	27 (33%)	22 (27%)	
	Preparation	32 (40%)	6 (7%)	
	Action	5 (7%)	17 (20%)	
	Maintenance	11 (13%)	11 (13%)	
Sleep	Pre-contemplation	38 (47%)	44 (53%)	0.711
	Contemplation	27 (33%)	22 (27%)	
	Preparation	16 (20%)	11 (13%)	
	Maintenance	0	6 (7%)	
Stress	Pre-contemplation	11 (13%)	28 (33%)	0.355
	Contemplation	27 (33%)	22 (27%)	
	Preparation	22 (27%)	6 (7%)	
	Action	16 (20%)	11 (13%)	
	Maintenance	5 (7%)	17 (20%)	
Social connectivity	Pre-contemplation	54 (67%)	66 (80%)	0.138
	Contemplation	22 (27%)	0	
	Preparation	5 (7%)	11 (13%)	
	Maintenance	0	6 (7%)	
Addictions	Pre-contemplation	3	1	-

Table 5 shows the adjusted ORs for lifestyle parameters associated with shift versus non-shift work among hospital support staff and nursing officers. Sleep quality was the only domain that showed a significant association among nursing officers, and non-shift workers were more likely to have better sleep quality (OR: 6.503; 95% CI: 1.106, 8.241; p = 0.038). However, no such association was found in hospital support staff. For flexibility, muscle endurance, cardio fitness and balance, no significant differences were observed between shift and non-shift workers in either group. No significant differences were found for stress or social connectivity in either group, although nursing officers had a higher, non-significant OR for stress (Table 5).

**Table 5:** Adjusted odds ratio for various lifestyle domain parameters associated with shift working pattern as compared to non-shift pattern in each job category Categorization for flexibility, muscle strength crunches, muscle endurance push, and cardio fitness - Poor: <L4, Good: ≥ L4; Flamingo balance test - Poor: Score > 0; Good: Score = 0; Sleep quality - Poor: PSQI > 5; Good: PSQI ≤ 5; Stress - More: PSS ≥ 19; Less: PSS < 19; Social connectivity - Poor: Score < 4; Good: Score ≥ 4. OR, odds ratio; CI, confidence interval. *After adjusting with age, sex, waist circumference, and whole body fat level.

Lifestyle domain	Job pattern	Job category (adjusted OR* [95% CI]; p-Value)	
		Hospital support staff	Nursing officers
Physical fitness			
Flexibility	Shift	1.000	1.000
	Non-shift	0.814 [0.499, 1.325]; 0.407	1.058 [0.847, 1.321]; 0.622
Muscle strength crunches	Shift	1.000	1.000
	Non-shift	-	-
Muscle endurance push	Shift	1.000	1.000
	Non-shift	1.084 [0.879, 1.338]; 0.451	1.187 [0.725, 1.944]; 0.495
Cardio fitness	Shift	1.000	1.000
	Non-shift	1.019 [0.755, 1.376]; 0.902	0.959 [0.573, 1.606]; 0.874
Flamingo balance test	Shift	1.000	1.000
	Non-shift	0.822 [0.624, 1.085]; 0.166	1.448 [0.924, 2.269]; 0.107
Sleep quality	Shift	1.000	1.000
	Non-shift	2.155 [0.194, 3.97]; 0.532	6.503 [1.106, 8.241]; 0.038
Stress	Shift	1.000	1.000
	Non-shift	6.929 [0.597, 80.379]; 0.122	9.015 [0.766, 106.05]; 0.080
Social connectivity	Shift	1.000	1.000
	Non-shift	0.338 [0.034, 3.409]; 0.358	1.273 [0.211, 7.680]; 0.793

Table 6 presents the multiple linear regression model showing the coefficients associated with total calorie intake. The model indicates that changing the job pattern from non-shift to shift significantly increases the mean total calorie intake by 536.2 units (p < 0.001). The hospital support staff had a higher mean total calorie intake compared to nursing officers, with the difference amounting to 101.69 units after adjusting for other confounding factors. Additionally, the mean total calorie intake for females was 442.29 units lower than that for males. Each unit increase in age reduces the mean total calorie intake by 23.27 units (Table 6). 

**Table 6:** Multiple linear regression model showing coefficients associated with total calorie intake *p-Value indicates statistical significance.

Parameter	Coefficient	Std. Error	t-Statistic	p-Value
Age in years	-23.267	18.200	-1.278	0.206
Sex	-442.291	145.076	-3.049	0.004*
Job pattern (non-shift)	536.200	138.437	3.873	<0.001*
Job category (nursing officer)	-101.697	149.912	-0.678	0.500

Table 7 shows the adjusted ORs for various lifestyle parameters associated with job categories (hospital support staff vs. nursing officers) within non-shift and shift work patterns. For flexibility and balance, nursing officers in the shift pattern had higher odds compared to hospital support staff, but the results were not statistically significant (p = 0.159 and p = 0.152, respectively). Similarly, for muscle endurance and cardio fitness, nursing officers showed no significant differences in either shift or non-shift patterns. Sleep quality and stress showed higher odds for nursing officers in the shift group than in the hospital support staff group, but these findings were not statistically significant (p = 0.367 and p = 0.150, respectively) (Table 7).

**Table 7:** Adjusted odds ratio for various lifestyle domain parameters associated with job category in each job pattern Categorization for flexibility, muscle strength crunches, muscle endurance push, and cardio fitness - poor: <L4, good: ≥L4; Flamingo balance test - Poor: Score > 0; Good: Score = 0; Sleep quality - Poor: PSQI > 5; Good: PSQI ≤ 5; Stress - More: PSS ≥ 19; Less: PSS < 19; Social connectivity - Poor: Score < 4; Good: Score ≥ 4. OR, odds ratio; CI, confidence interval. *After adjusting for age, sex, waist circumference, and whole body fat level.

Lifestyle domain	Job category	Job pattern (adjusted OR* [95% CI]; p-value)	
		Non-shift	Shift
Physical fitness			
Flexibility	Hospital support staff	1.000	1.000
	Nursing officers	1.352 [0.144, 12.681]; 0.792	6.827 [0.471, 99.004]; 0.159
Muscle strength crunches	Hospital support staff	1.000	1.000
	Nursing officers	-	-
Muscle endurance push	Hospital support staff	1.000	1.000
	Nursing officers	1.056 [0.133, 8.383]; 0.959	1.757 [0.195, 15.875]; 0.616
Cardio fitness	Hospital support staff	1.000	1.000
	Nursing officers	0.125 [0.001, 22.008]; 0.431	0.497 [0.061, 4.022]; 0.512
Flamingo balance test	Hospital support staff	1.000	1.000
	Nursing officers	0.725 [0.124, 4.223]; 0.720	7.239 [0.484, 108.284]; 0.152
Sleep quality	Hospital support staff	1.000	1.000
	Nursing officers	1.382 [0.160, 11.902]; 0.768	2.438 [0.352, 16.867]; 0.367
Stress	Hospital support staff	1.000	1.000
	Nursing officers	0.920 [0.154, 5.494]; 0.927	6.163 [0.519, 73.414]; 0.150
Social connectivity	Hospital support staff	1.000	1.000
	Nursing officers	1.461 [0.203, 10.535]; 0.707	7.154 [0.586, 87.297]; 0.123

## Discussion

This study aimed to assess the impact of shift work on the various physiological and lifestyle characteristics of hospital workers and nursing officers.

Across both hospital workers and nursing officers, shift workers demonstrated significantly higher body weight and waist circumference than their non-shift counterparts, which is consistent with previous research linking shift work to weight gain and increased central adiposity [3,11-13]. These changes in body composition may be attributed to the circadian misalignment disrupting the body’s natural rhythms, directly impacting metabolism and hunger hormones. It reduces leptin, the hormone that suppresses appetite and increases ghrelin, which stimulates hunger. This imbalance can lead to overeating, weight gain, and metabolic disorders over time [14]. In addition, shift workers were found to consume significantly more calories than non-shift workers in both groups, further supporting the idea that shift work contributes to unhealthy eating behaviors [14]. Water intake was notably lower in shift workers across both tables, which could be due to the limited opportunities to hydrate during shifts. Hydration is crucial for maintaining metabolic balance and energy levels, and its neglect among shift workers could compound the negative health effects of their job schedules. Cardiovascular fitness was significantly lower in shift workers, particularly among nursing officers, who also exhibited higher resting metabolism, likely reflecting the physical demands of their profession combined with the physiological stress of irregular work hours. Lower cardiovascular fitness in shift workers has been linked to circadian disruption and increased stress, both of which negatively affect heart health [15]. Sleep quality is a major area of concern for shift workers, with significant differences in the PSQI scores among nursing officers, indicating poorer sleep among shift workers. Although the total hours of sleep were not significantly different, the lower quality of sleep in shift workers may be a consequence of circadian misalignment and the challenges of maintaining consistent sleep patterns during rotating or night shifts [16]. Poor sleep exacerbates other health risks, such as weight gain, reduced cardiovascular fitness, and increased stress [17]. Interestingly, while shift workers experienced higher stress levels in the general worker population, nursing officers in shift jobs reported lower stress levels. This paradox could be explained by different coping mechanisms or workplace demands in nursing, where shift work is normalized and potentially perceived as less stressful in comparison to other non-medical staff roles. This study emphasizes the importance of customized interventions addressing nutrition, sleep, physical activity, and mental health for shift workers, particularly nursing officers. Furthermore, policy reforms and workplace modifications tailored to healthcare providers are crucial for reducing long-term health risks and enhancing workforce well-being.

This study found no significant differences in readiness to change lifestyle behaviors between shift and non-shift workers across various domains, including nutrition, physical activity, sleep, stress, social connectivity, and addictions. Both groups showed similar patterns of readiness, with many participants in the pre-contemplation stage, particularly for nutrition and sleep. Despite evidence suggesting that shift workers may face more challenges due to irregular schedules [18,19], our results indicate that motivation to change lifestyle habits is not markedly different from that of non-shift workers. These findings suggest that the type of work (shift vs. non-shift) may not be a major factor influencing readiness for lifestyle change. The pre-contemplation stage signifies that individuals are not yet considering or ready to make changes to their lifestyle behaviors. They may be unaware of the need for change, lack motivation, or feel resistant due to perceived barriers. This highlights the importance of awareness-building initiatives, motivational counseling, and targeted interventions to help shift workers recognize the benefits of adopting healthier habits. Workplace health programs should focus on supporting sustainable behavior changes rather than solely targeting work schedules.

This study also found that shift work significantly affected sleep quality among nursing officers, with non-shift workers being more likely to have better sleep quality (OR: 6.503, p = 0.038). This aligns with the known disruption of circadian rhythms caused by shift work, especially in demanding healthcare roles [8-11]. However, no significant differences in sleep quality were found among hospital support staff, possibly because of differences in job stress levels. For other lifestyle parameters, such as flexibility, muscle endurance, cardio fitness, and balance, no significant associations were observed between shift and non-shift workers in either group, suggesting that physical fitness may not be directly influenced by work patterns. Although stress and social connectivity did not show significant differences, nursing officers in non-shift work had a higher, non-significant OR for stress, indicating a potential link that requires further investigation.

The findings from the multiple linear regression analysis reveal key factors influencing total calorie intake among workers. A significant association was found between job patterns and calorie intake, with non-shift workers consuming an average of 536.2 more calories than shift workers did (p < 0.001). This suggests that shift workers may have altered eating habits or reduced calorie consumption, possibly due to irregular work hours affecting their meal patterns. In terms of sex, females were found to consume 442.29 fewer calories on average than males, which is consistent with previous studies showing lower energy intake in females than in males. However, this difference could also be influenced by physiological and lifestyle factors which were not explored in the current model [20].

Although the shift from general workers to nursing officers was associated with a reduction in calorie intake (by 101.69 units), this result was not statistically significant, indicating that job category alone may not be a strong determinant of dietary behavior. Additionally, while age showed a decreasing trend in calorie intake (by 23.27 units per year), this relationship was not significant, possibly reflecting individual variability in dietary needs as people age.

This study found no significant associations between job category (hospital support staff vs. nursing officers) and lifestyle parameters within shift and non-shift work patterns, likely due to the small sample size. While shift nursing officers showed higher odds of better flexibility, balance, sleep quality, and stress management, these trends were not statistically significant. These results suggest that job category and work pattern may not strongly impact lifestyle outcomes. Future studies with larger sample sizes are required to clarify these relationships.

The primary limitation of this study is the small sample size, which may have reduced the ability to detect statistically significant differences in some lifestyle domains. Additionally, the study’s design limits its ability to establish causality between shift work and lifestyle changes; however, it provides a snapshot in time. Furthermore, self-reported data on calorie intake and physical activity may have introduced reporting bias. Future research should aim for larger sample sizes and longitudinal designs to better understand the long-term impact of shift work on health and lifestyle. Further studies should explore specific interventions targeting nutrition, hydration, and physical activity to improve the well-being of shift workers.

## Conclusions

This study highlights the significant impact of shift work on various lifestyle and health parameters among hospital workers. Shift workers, particularly nursing officers, exhibited higher body weight, increased calorie intake, poorer cardiovascular fitness, and lower sleep quality than their non-shift counterparts. Despite these trends, readiness to change lifestyle behaviors was similar between shift and non-shift workers, suggesting that work patterns alone may not be the primary driver of lifestyle changes. These findings emphasize the need for targeted interventions to address health risks associated with shift work, particularly focusing on nutrition, sleep hygiene, and cardiovascular fitness. The study’s findings could extend to workers in other industries with comparable shift patterns, warranting further investigation.
